# The Implementation of the Professional Role of the Community Pharmacist in the Immunization Practices in Italy to Counteract Vaccine Hesitancy

**DOI:** 10.3390/pharmacy8030155

**Published:** 2020-08-25

**Authors:** Rosagemma Ciliberti, Nicola Luigi Bragazzi, Alessandro Bonsignore

**Affiliations:** 1 Section of History of Medicine and Bioethics, Department of Health Sciences (DISSAL), University of Genoa, 16132 Genoa, Italy; rosellaciliberti@yahoo.it; 2 Postgraduate School of Public Health, Department of Health Sciences (DISSAL), University of Genoa, 16132 Genoa, Italy; 3 Laboratory for Industrial and Applied Mathematics (LIAM), Department of Mathematics and Statistics, York University, Toronto, ON M3J 1P3, Canada; 4 Section of Legal and Forensic Medicine, Department of Health Sciences (DISSAL), University of Genoa, 16132 Genoa, Italy; alessandro.bonsignore@unige.it

**Keywords:** pharmacovigilance and vaccinovigilance, vaccine hesitancy, immunization programs, vaccination, pharmacists, health services accessibility

## Abstract

In Italy, the National Vaccinal Prevention Plan has renewed the commitment of the Italian government to promote a culture of vaccination practices in the general population and especially among healthcare professionals, considering it as a strategic goal. The search for useful tools and techniques to promote a layered and widespread information network capable of restoring a climate of trust and confidence towards vaccination, leads us to reflect on the possibility, already adopted in numerous countries, of enlisting community pharmacies in immunization campaigns also in Italy, positively implementing the professional role of the community pharmacist in immunization. The pharmacist is often the first point of contact with both the patients and the public, both for the relationship of trust and confidence that binds him to the citizens, and for the ease of access in relation to the widespread distribution of community pharmacies in the territory, the availability of prolonged operating hours, the absence of need for appointments and positions near/outside of healthcare facilities. Currently, in Italy the role of the community pharmacist is limited to counseling and providing advice and information regarding the benefits and/or any risks of vaccination practices, but does not imply a direct engagement in immunization programs, rather a collaboration to avoid straining and overwhelming the vaccination centers. Some recent questionnaire-based studies have shown that Italian community pharmacists have attitudes that are favorable to vaccinations, even though their knowledge is rather limited. Together with expanding the engagement of community pharmacists in immunization programs, their educational gap should be addressed in order to significantly improve and enhance the protection of the public health.

## 1. Introduction

Infectious diseases impose a relevant burden, both from an economic and societal perspective [1,2]. The implementation of immunization practices has, therefore, represented a major turning point in the history of healthcare systems, making is possible to significantly protect, improve public health and prevent up to 2.5 million deaths every year worldwide [1]. The increase in life expectancy recorded over the past decades, closely related to the sharp reduction in mortality caused by communicable diseases, is certainly attributable to a significant extent to vaccination measures [3].

Vaccinations are considered among the most efficacious and cost-effective public health interventions [4]. However, in the 1990s, alleged non-evidence-based safety concerns in some countries have led to a fall in the vaccination coverage rate, causing the re-emergence of some infectious diseases, including polio, with various clusters and outbreaks in several Eastern European states, which were promptly contained thanks to extensive vaccination campaigns [5].

Alleged associations, even though authoritatively denied, between vaccination and autism [6] together with some judgments that, improperly, indicated a causal relationship between a case of autism in a child immunized with the hexavalent vaccine have represented the beginning of the so-called “vaccine hesitancy” [6,7,8,9]. This expression refers to a broad spectrum of phenomena, that range from delay in vaccine acceptance, uncertainty or indecision to frank reluctance, skepticism and refusal/rejection. Vaccine hesitancy is a complex, multi-factorial concept because it is shaped by various contextual determinants, such as the geographical area, the local organization, available resources, and the historical period, among others, as well as by individual and group variables. Moreover, vaccine hesitancy can be vaccine-specific or regard vaccinations in general: it represents a challenging concern, in that it can seriously jeopardize global health.

The growth of this phenomenon in Europe as well as in other socially advanced countries involves heterogeneous populations, which express their indecision or fear towards specific vaccines in a different way, whereas others reject all vaccine products without any distinction. In this context of growing suspicion, in Italy the withdrawal of two batches of an anti-influenza (Fluad) vaccine carried out prudently and in a precautionary manner by the National Medicines Agency (“Agenzia Italiana del Farmaco”, AIFA) in relation to three deaths that occurred within 48 h after the immunization, has certainly further reduced the public’s trust towards vaccine prophylaxis safety and effectiveness. Subsequent investigations conducted by the Higher Institute of Health (“Istituto Superiore di Sanità”, ISS), by the Pharmacovigilance Risk Assessment Committee (PRAC), as well as by the European Medicines Agency (EMA) Committee have excluded any causality between the reported deaths and the vaccine administered [10,11,12,13].

However, the media impact regarding the decision taken by AIFA has led to a significant reduction in the vaccination coverage rate against influenza when compared to previous years [10,11,12,13]. Studies indicate that awareness of risks related to infectious diseases is an important determinant when deciding whether to vaccinate or not [14], but mainstream opinion and social media can also greatly influence public opinion and awareness towards emerging/re-emerging infectious diseases [15].

The dramatic drop in vaccinations led Italy to record the highest number of measles cases in Europe in 2014 [13,16]. The same World Health Organization (WHO) has explicitly advised our country to take serious measures in this regard.

The general breakdown of the relationship of trust and confidence between the public institution, the scientific community and the citizens has, moreover, contributed to intensifying the complex and articulated debate (with respect to the different actors/stakeholders involved and to variegated positions expressed in the choice of vaccinating a child or not) between supporters and opponents of immunization practices [17], often reductively represented by the mass media in a rigid dichotomy (“pro-vax” versus “no-vax”), even though, as previously mentioned, the phenomenon of vaccine hesitancy is rather a continuum between these two extremes.

The debate worsened, particularly with the enactment of the law decree number 73/2017 containing urgent provisions on vaccine prevention, which made the following vaccinations compulsory and free of charge for minors between the ages of zero and sixteen: (a) poliomyelitis; (b) diphtheria; (c) tetanus; (d) hepatitis B; (e) pertussis; (f) *Haemophilus influenzae* type b; (g) meningococcal B; (h) meningococcal C; (i) measles; (j) rubella; (k) mumps; (l) chickenpox [18].

The same type of regulatory source used by the Italian government, the “decree-law”, is strongly expressive of the preconditions of “extraordinary necessity and urgency” (expressly stated in the preamble of the decree) and the willingness to intervene promptly to maintain adequate conditions for the epidemiological safety in terms of prophylaxis and vaccination coverage on the national territory, as well as “guaranteeing compliance with the obligations assumed and the strategies agreed at European and international level and with the common objectives set in the European geographical area”. On these premises, the article number 1 of the law n.73/2017 provides, for minors up to sixteen years of age, twelve compulsory and free vaccinations (even though in converting of the decree into law, mandatory vaccinations have actually been reduced to 10). Of these, eight (anti-pertussis, *Haemophilus influenzae* type B, meningococcal type B and C, measles, rubella, mumps and chicken pox) were not required by the previous legislation. The obligation is also excluded in the event of immunization following a natural disease, as well as in the presence of an ascertained danger to health in relation to specific clinical conditions, to be documented in the ways established by the same article number 1 of the decree.

The measure therefore responds to the criticisms and allegations related to the decrease in vaccination coverage, compared to the optimal threshold of 95% as recommended by the WHO for achieving the so-called “herd immunity”. The 2017–2019 National Vaccine Prevention Plan reveals, in fact, that while vaccination coverage has grown to stabilize until the middle of the first decade of 2000, reaching 95 per cent, but not for measles, mumps and rubella, the trend is reversed in the most recent period, given the data referring to 2015 and concerning the 2013 cohort, which confirm the trend of decline already underway in the previous three years. The legislation has, however, reopened a heated debate on the vaccination strategy based on coercion rather than conviction [16].

In rejecting all the objections of constitutional legitimacy on the Italian law decree, the Italian Constitutional Court with the sentence number 5/2018 highlights the enhanced value of the contributions and opinions expressed by qualified public authorities. These include the WHO, the Italian National Institute of Health, the Council of the National Federation of Orders of Surgeons and Dentists (FNOMCeO), the National Academy of Lincei (“Accademia Nazionale dei Lincei”) and four scientific and professional associations (the Italian Society of Hygiene, Preventive Medicine and Public Health, “Società Italiana di Igiene Medicina Preventiva e Sanità Pubblica” or SITI; the Italian Society of Pediatrics, “Società Italiana di Pediatria” or SIP; the Italian Federation of Pediatricians, “Federazione Italiana Medici Pediatri” or Fimp; and the Italian Federation of General Practitioners, “Federazione Italiana Medici Di Medicina Generale” or FIMMG). These organisms are highly active with specific publications and proposals in the sector of vaccination policy. Despite the variety of settings, all the documents stigmatized the decline in coverage and recommended greater commitment and responsibility in the promotion of vaccinations against preventable diseases. As regards the ethical profiles, the decision of the Italian judge refers to the motion “The importance of vaccinations”, approved unanimously on 24 April 2015 by the National Committee of Bioethics (“Comitato Nazionale di Bioetica”, CNB), which underlines the need to make all possible efforts to achieve and maintain optimal vaccination coverage through public education programs and health workers, not excluding mandatory measures during emergencies (such as outbreaks). The Italian Constitutional Court also appreciates the so-called “flexibility clause” or “necessary update”, which provides a mechanism to ease the degree of compulsion of the vaccine obligation in relation to the updating of the data that progressively emerge in the appropriate scientific forums, explicitly expressing that the legislative choice in favor of the obligation instrument is strongly anchored to the context and is susceptible of possible different evaluations.

In line with the strategic guide provided by the European Vaccine Action Plan 2015–2020 (EVAP), the Italian National Vaccinal Prevention Plan (PNPV) 2017–2019 has renewed the governmental commitment to promote a culture of vaccination practices in the general population and especially among healthcare professionals, considering it as a strategic goal.

The search for useful tools and techniques to promote a layered and widespread information network capable of restoring a climate of trust and confidence towards vaccination, leads us to reflect on the possibility, already adopted in numerous countries, of enlisting community pharmacies in immunization campaigns also in Italy, positively implementing the professional role of the community pharmacist in immunization [19].

The pharmacist is often the first point of contact with either the patients and the public, both for the relationship of trust and confidence that binds him to the citizens, and for the ease of access in relation to the widespread distribution of community pharmacies in the territory, the availability of prolonged operating hours, the absence of need for appointments and positions near/outside of healthcare facilities.

The community pharmacist is also often in a privileged position both of dialogue with the population, in relation to the constant attendance and usage of the services he provides, and the great level of confidence and trust that can be created in an ongoing relationship. Moreover, the community pharmacist can help in the identification of people who present particular risk factors or unhealthy lifestyles and can provide educational and counseling activities, providing patients with evidence-based facts, addressing common misconceptions about vaccinations and potentially preventing future outbreaks of vaccine-preventable diseases [19,20].

## 2. The Role of the Community Pharmacist in Immunization Practices: An International Perspective

Since the middle of the 19th century, community pharmacists have always played a key role in immunization practices through the distribution of smallpox vaccine. Even in difficult periods such as the First World War, community pharmacists made an important contribution in educating doctors and potential patients on vaccines.

In 1994, the Washington State Pharmacists Association initiated the first ongoing formalized training of pharmacists in vaccine administration. In 1994, the USA Department of Health and Human Services (DHHS) officially recognized the important contribution of the USA community pharmacists to the immunization process in relation to the needs of education, distribution, administration, registration and monitoring of vaccine products. On 1 November 1996, the American Pharmaceutical (now Pharmacists) Association (APhA) began its nationally recognized training program for community pharmacists, known as the Pharmacy-Based Immunization Program. During the second half of 2009, the spread of the influenza A-H1N1 virus in the USA led many American states to allow community pharmacists to administer vaccinations together with other healthcare workers. According to the position paper published in 2018 by the Pediatric Pharmacy Advocacy Group (PPAG), in 48 out of 52 USA states (including Puerto Rico and Washington, DC, USA), community pharmacists are permitted to administer any vaccine, with the four remaining states allowing, instead, limited vaccine administration [21,22].

In Argentina, the community pharmacist has been authorized to inoculate most of the immunization antigens and vaccine products (hepatitis B, *Haemophilus influenzae* and the trivalent diphtheria/tetanus/pertussis (DTP) vaccine, etc.) since 1983 [22].

In 2016, the International Pharmaceutical Federation (FIP) has prepared a report resulting from a large survey conducted in 45 countries. The Report has shown that up to 20 countries provide citizens with the possibility of obtaining at least one vaccination in community pharmacies, and, in 13 countries (namely, Argentina, Australia, Canada, Costa Rica, Denmark, Ireland, New Zealand, the Philippines, Portugal, South Africa, the United Kingdom, the United States and Switzerland), community pharmacists with specific training requirements can administer mandatory vaccines alone or in collaboration with other qualified allied health professionals directly in the community pharmacy.

Furthermore, other seven countries (namely, Bolivia, Democratic Republic of the Congo, Finland, Iceland, Lebanon, Holland and Pakistan) are ready to join this list. In Canada, in the province of Québec, nurses working in community pharmacies are allowed to administer not only the flu vaccination, but also vaccines against hepatitis A and B and others intended for the prophylaxis of travelers [23].

Among European countries, France has also recently started to allow community pharmacists to practice vaccination against seasonal flu directly in pharmacies, with certain requirements and professional skills acquired through specific staff training courses [24].

However, in 2019, Poudel et al. [25] highlighted a very varied international picture of the role of community pharmacists in vaccination strategies and practices. Significant differences concerning the type of service offered, their training, the type of vaccine authorized for administration, the type of population admitted to this service, the type of intervention and the economic conditions were also recorded within the same country with consequent obstacles to implementing an effective vaccination program. On the social and economic-financial side, in 2018, Bartsch et al. [26] showed that adding community pharmacists as vaccinators in immunization campaigns could avoid up to 11.9–16.0 million cases of flu, and 23,577–210,228 deaths, with relevant savings of $1.0–2.8 billion in direct costs and $4.1–99.8 billion in total costs.

## 3. The Role of the Community Pharmacist in Immunization Practices in Italy

In Italy, the professional figure of the community pharmacist plays, in cooperation with all the other allied health professionals, a significant role in delivering and divulging evidence-based, correct information, monitoring and surveillance of adverse reactions to drugs. Furthermore, it is of paramount importance in the prevention/mitigation of the burden generated by infectious and diffusive diseases through mandatory, proposed/recommended or strongly recommended vaccine prophylaxis practices to the population that are at developmental age and that are at risk in our country, respectively [27]. It should be, anyway, emphasized that in Italy community pharmacists are not directly involved in vaccine administration.

This role, particularly significant in the current context of progressive reduction in vaccine compliance, is in fact already explicitly described in the legislative decree number 153 of 3 October 2009, “Identification of new services provided by pharmacies within the National Health Service, as well as provisions on residence allowances for owners of rural pharmacies, in accordance with the article number 11 of the law of 18 June 2009, number 69”. This decree, indeed, implies “the collaboration of pharmacies in initiatives aimed at guaranteeing the correct use of prescribed and dispensed medicines and their monitoring, at promoting the patient’s adherence to medical therapies, also through participation in specific pharmacovigilance programs” (article number 1, point number 2) [28].

The information/training role of pharmacists is also underlined by the commitment of community pharmacies to participate in the implementation of health education programs and prevention campaigns for the main pathologies with a strong societal impact, targeting either the general population and at-risk groups. In addition, the legislation expressly provides (article number 1, paragraph number 5) that the National Health Service (NHS) itself promotes the inter-professional collaboration of community pharmacists from public and private pharmacies operating in agreement with the NHS with general practitioners and free-choice pediatricians, in reference to the activities of the new aforementioned services.

The role of the community pharmacists in informing, advising, counseling and promoting/enhancing public health is also evident in numerous provisions of the 2018 Italian Pharmacist Code of Conduct (“Codice Deontologico del Farmacista”, CDF), which establishes the obligation of the community pharmacist, being a public health care worker:

- to collaborate with the authorities, assisting them in achieving institutional objectives;
- to participate in prevention and health education campaigns promoted or organized by the competent Authorities in agreement with the National Federation of the Orders of Pharmacists or with the territorial/local Order;
- to contribute to the protection of public health through a precise observance of the rules;
- to be engaged in pharmacovigilance/vaccinovigilance programs;
- to provide clear, correct and complete health information in professional advice and counseling, with particular reference to the appropriate use of the medicines, their contraindications, side effects and their effectiveness.

On the basis of these provisions, the community pharmacist’s commitment concerns properly informing the public about the benefits of immunization, the contraindications and possible side effects of vaccinations, eliminating prejudices and promoting adherence to the vaccination schedule.

Moreover, the provisions of the Guiding Principles indicated in the PNPV 2017–2019 specify that: “Every healthcare worker, or whoever carries out tasks for any reason on behalf of the National Health Service, is ethically obliged to inform, advise and promote vaccination practices in accordance with the most up-to-date scientific evidence and shared strategies at the national level” (point number 5 “Ethics”).

Various deontological provisions also establish duties of collaboration and cooperation with other allied health professionals. It is also appropriate to emphasize that the provisions contained in the CDF are mandatory and binding in order to guarantee the health of the citizens, the community, as well as to protect the ethics and the dignity of community pharmacists and other allied health professionals. Failure to comply with these rules, such as the dissemination of misleading information, is therefore not only ethically unacceptable, but constitutes a serious infringement of the professional ethics and it is, as such, contractually and legally punishable by disciplinary actions.

Currently, in Italy the role of the community pharmacist is limited to counseling and providing advice and information regarding the benefits and/or any risks of vaccination practices, but does not imply a direct engagement in immunization programs. Recently, the President of the National Council of the Federation of the Orders of Italian Pharmacists (“Federazione Ordini Farmacisti Italiani”, FOFI) has declared his adhesion to the possibility of expanding the role of community pharmacists in vaccination campaigns, in order to increase vaccine uptake and coverage rate. In more detail, in its report, the FOFI has also specified that immunization would not be practiced directly by the community pharmacist, but by authorized personnel only, offering however a direct, mutual collaboration to avoid straining and overwhelming the vaccination centers.

This collaboration between different public health workers and allied health professionals, fully in line with the provisions of the CDF, can represent the key to obtaining other high quality results in the delivery of healthcare provisions, such as the active surveillance and monitoring of adverse reactions potentially related to vaccine products (“vaccinovigilance”) and constitutes a good practice to be constantly strengthened from the training phase of the community pharmacists onwards.

More specifically, from the point of view of public ethics, the current legal obligation of vaccinations implies, moreover, the commitment of public health facilities to guarantee all the conditions to facilitate the implementation of the vaccination practice starting from the educational commitment, in the best interest of the common good.

A very recent experiment launched in 2020 in Carnia [29], a mountainous region of northern Italy where the NHS is more critical, showed that emphasizing the role of community pharmacies and involving them in order to strengthen the health network and bring the Regional Health System (“Sistema Sanitario Regionale”) closer to the citizen have led to an increase by 5.2% of the vaccination coverage rate compared to the previous year, which represents, in terms of percentage variation, the best increase in the region (versus an average of 2.2%). The initiative concerned the distribution of drugs and food for cancer patients, and the strengthening of adherence to the vaccination campaign. The objective of the region is to extend the initiative also to over 400 regional pharmacies or “health points” spread throughout the territory, as indicated in the health reform law, which can contribute to providing proximity services together with general practitioners and free-choice pediatricians, in addition to distributing drugs, medical devices and promoting health, particularly for the elderly, frail and at-risk subjects. Specifically, the planning of the anti-flu campaign, launched by the prevention department with the pharmaceutical service together with Federfarma and with 25 local pharmacies last October, is part of the agreement signed between the local health unit and the community pharmacies for the promotion of services for the local population.

However, the current legislation (Consolidated Health Laws, R.D. 27/07/1934, n. 1265, article number 102l) still prevents community pharmacists from carrying out some “medical” activities. Similarly to what happened in other countries, the attempt to involve pharmacists in the vaccination intervention has encountered strong opposition from medical associations. For instance, the FNOMCeO has expressed its opposition to a change in the current legislation, expressing fear in the traceability of responsibilities, concerns regarding insurance coverage and disorientation among healthcare professionals. Equal doubts and resistances have also been registered by the Italian Family Physicians Federation (FIMMG) which has underlined the possible “dangerous relationships” in the performance of “medical” activities at community pharmacies. In line with these associations, the Italian Federation of Pediatricians (Fimp) also stressed that vaccination is not a mere inoculation, but requires professional skills, training, sensitivity, organization, relationship skills, and ease of access, which families can find only in the context of primary care and the widespread organization that family pediatricians and general practitioners offer in Italy. The FOFI has replied to these disputes, underlining the different involvement that the Italian community pharmacist would have in the implementation of the immunization programs, which would in any case be entrusted, compared to what can be seen in other countries (for example, Britain), to the medical doctors.

Within this framework, Scarpitta et al. [30] devised an online, standardized, self-administered and validated “knowledge and attitudes” questionnaire, consisting of five sections and 28 items. The questionnaire was distributed to a sample of 120 community pharmacists working in Western Sicily. Most of them (99.2%) agreed with the Regional Vaccination Schedule and 95% were fully vaccinated and in favor of immunization practices, having already vaccinated, or being willing to vaccinate in the future, their children. Up to 90% of their clients asked for information about vaccinations. Citizens’ trust and confidence towards vaccination practices were found to have increased (30%) or remained stable (54.2%) in the last five years, being positively influenced by evidence-based, high-quality counseling provided by family pediatricians and general practitioners and negatively by misinformation divulged by mass-media and web-sites.

However, despite such favorable attitudes, there are some gaps in community pharmacists’ education towards vaccinations. Della Polla and colleagues [31] carried out a cross-sectional study based on a “knowledge, attitudes, and behaviors” (KABs) questionnaire. This was distributed to a sample of 389 community pharmacists. Knowledge concerning mandatory vaccinations was limited with only 23.9% of interviewees indicating correct replies. In terms of practices, most of them (91.7%) believed that community pharmacists can play a key role in immunization programs, by providing proper information, and 75.3% would like to be more engaged in educational activities related to vaccine interventions. On the other hand, less than one third (23.7%) had recommended anti-human Papillomavirus (HPV) vaccine to adolescents aged 11–12 years old.

Similarly, Della Polla and coworkers [32] highlighted further gaps in community pharmacists’ education concerning infectious diseases and vaccinations. One of these gaps regards travel medicine and related infections. Once again, knowledge was rather limited, with only 1.8% of the interviewees answering all seven knowledge questions correctly. The mean knowledge score was 2.9 out of 7, or around 41% of items answered correctly. On the other hand, more than two-thirds of the interviewees believed that community pharmacists could play a prominent role in properly informing the public about travel medicine and related infectious disorders and, actually, they often or always provided such information and/or referred to a travel health center for counseling, when appropriate.

## 4. The Role of Communication and Information

The access to information–informed consent constitutes a general prerequisite for the guarantee of general fundamental rights, also unrelated to the specific area concerning the protection of health [33,34].

The lack of correct, scientifically validated, reliable and verifiable information can seriously jeopardize and impact the meaning and scope of the principle of self-determination as it is capable of affecting the formation of an authentically aware conviction. The possibility of conveying information not all verifiable by origin and truthfulness through information tools and social networks that have a strong accessibility and dissemination is therefore an important critical factor. On the other hand, in the presence of information distortions/biases or, in any case, of communication inadequacies, the strategies based on the recommendation alone may not be completely effective due to the failure to achieve an adequate coverage rate suitable to guarantee indirect benefits to the entire population, particularly fragile subjects (elderly and/or individuals suffering from infectious and chronic-degenerative pathologies).

Moreover, and especially in the field of vaccine prophylaxis, carefully planned information, based on confirmed evidence and data and involving the involvement of all stakeholders (doctors, health workers, and patients), can play a crucial and decisive role, particularly for the subjects who should get vaccinated and for the subjects unable to express an autonomous consent. In 2018, Pelullo and Di Giuseppe [35], in a study conducted on an Italian sample of 771 adolescents aged 11 to 18 years, showed how a more favorable attitude by adolescents towards vaccination intervention was significantly associated with received information, highlighting the need to implement educational interventions to improve adolescents’ understanding of vaccinations in order to provide them with greater awareness in their health decisions. The conscious use of medicines, particularly those over the counter that are freely accessible without the filter of adults or health professionals, but also the use of contraceptive systems and protection of sexual health, constitute opportunities for precious interaction that teenagers can have with community pharmacists as easily accessible interlocutors to involve this vulnerable population in conversations on vaccine prophylaxis.

In 2017, Abraham et al. [36] highlighted that USA adolescents often declare their interest in talking with community pharmacists about drug-related topics and have expressed interest in the knowledge provided by pharmacists, in particular on issues related to the influence of drugs on the body, their interaction, their production, new emerging studies and accumulating evidence. At the same time, missed vaccination opportunities occur when there is contact with health services, but this has not resulted in the vaccination practice in a patient eligible to receive the necessary vaccines. This aspect is particularly significant in adolescents among whom vaccination opportunities decrease in relation to the fact that, at this stage of development, they are no longer subject to careful monitoring by pediatricians and often do not require ongoing medical treatments [37,38,39].

In order to promote and enhance greater awareness of the role of vaccination campaigns and self-care culture as a tool for prevention, health and well-being, the National Federation of Pharmacy Holders (FEDERFARMA) has declared the full availability of community pharmacies as regards both the organization and implementation of effective vaccination campaigns and the distribution of vaccines, as well as the commitment to raising awareness among citizens in the face of growing disaffection towards immunization practices [40].

The need to provide, in exceptional situations due to the alert for the documented drop in vaccination coverage rate below the threshold necessary to achieve herd immunity, must also be combined with the continuous commitment to identify and implement all of the most appropriate initiatives aimed at promoting and implementing the persuasiveness approach as an optimal model, not only to bring the relations between the State and regions in this material sphere within the ordinary tracks, but, even more, from an ethical point of view.

## 5. Conclusions: Past, Present and Future

The progressive reduction in trust and confidence towards vaccination and the decrease in the adherence and compliance levels to vaccine prophylaxis measures, with the consequent drop in the active immunization rate, urge the adoption of public health initiatives and interventions aimed at promoting a culture of personal and societal responsibility towards vaccination programs. The integration of community pharmacy services into national vaccination programs, in a complex, articulated and multidisciplinary approach, could help raise awareness among the population, particularly at-risk and vulnerable categories, by promoting the uptake of vaccine and increasing the vaccination coverage rate in Italy, as well as in the European Union, counteracting the spread of unreasonable prejudices and skepticisms.

In order to meet the primary public health protection needs at the population level, it is of paramount importance to establish interactions with all the actors and stakeholders involved in the immunization practices, including general practitioners and free-choice pediatricians. Shared paths paving the way for the promotion and development of vaccination campaigns in pharmacies should be designed and implemented. In several European Union countries, such as Denmark, Ireland, Portugal, the UK, and Switzerland, this is already a reality.

It should be stressed that this vision of public health is characterized by complementarity and not by competitiveness between the various professional figures that together work for a common objective, the enhancement of public health. Only in this way it would be possible to really increase vaccination coverage rates.

Community pharmacists can offer their expertise in terms of relational, social, management and organizational skills to make immunization campaigns successful and effective. Community pharmacists are territorial health services characterized by high accessibility and capillarity. Pharmacists can interact with physicians, general practitioners and other healthcare workers, immunizing and spreading positive, reliable information on vaccination. Another great advantage of the “community pharmacist as immunizer” is represented by the opening hours of pharmacies, which can facilitate both workers and non-workers and is particularly important in rural, isolated and underserved areas, where access to vaccination points as well as to other healthcare services and facilities can be very challenging.

Replying to the various criticisms and oppositions to making immunization campaigns mandatory may prove to be insufficient. More specifically, from the point of view of public ethics, the current legal obligation of vaccinations implies, moreover, the commitment of public health facilities to guarantee all the conditions to facilitate the implementation of the vaccination practice, starting from the educational commitment, in the best interest of the common good.

Widening dialogue, promoting information and developing a critical conscience can better provide citizens with all the elements necessary to correctly understand the importance of vaccinations, both at individual and collective levels, underlining a peculiar characteristic of vaccinations, which is their high societal value. The multi-factorial nature of the obstacles to vaccination (biological, social, economic, logistic and epidemiological) requires the adoption and implementation of broad, multi-component and differentiated strategies. On the other hand, the role of the healthcare team which, in Italy, has had a formal and explicit recognition with the aforementioned law 219/2017 on the subject of informed consent and advance treatment provisions, calls for shared strategies and new systems of collaboration and coordination that, in compliance with the specific skills and professionalism of the various operators, can corroborate the health protection system.

A further aspect regards the possibility of positively increasing the collaboration between the various allied health professionals and public health operators, both through the possibility of strengthening the training on vaccinations in the medical and pharmacy study plans, and the possibility of providing common training courses that can favor the adoption of synergies in order to improve and enhance the protection of public health.

These new commitments should be supported by means of the implementations of logistical and organizational reforms, from a networking of initiatives and actions that can really favor the functioning of a territorial team comprising various health care workers, allied health professionals and other relevant actors and stakeholders, including community pharmacists, without the perception of undue invasions in the field.

Finally, changes in training and education, as well as in the legislation and allocation of adequate funding to recognize the increased scope of practice of community pharmacists, are absolutely crucial, in that they would benefit the entire health system and citizens’ health.

Recognizing access to health records is also important. Community pharmacists should be able to access vaccination records, including details of the prescriber, patient name, type of product injected and route/method of administration.

All these issues are crucial, especially considering the currently ongoing “Coronavirus disease 2019” (COVID-19) outbreak [41], which has uncovered new opportunities for community pharmacists, who have played a key role during the emergency. They have promptly and quickly responded to the pandemic, ensuring the drug supply chain and guaranteeing continuity of care, providing tele-pharmacy and tele-health consulting services, reporting and referring COVID-19 cases, taking part into clinical experimentations and trials, divulging high-quality COVID-19-related information and addressing health inequity, besides delivering classical prescribing services. All of this suggests that fully integrated “medical-pharmaceutical networks” and a multi-layered, inter-sectoral and inter-professional collaboration can help better tackle crises and public health emergencies. It can be expected that community pharmacists will play a major role in late Fall/Winter 2020, during the yearly immunization campaign against influenza. Since non-pharmacological interventions implemented to mitigate against COVD-19 (such as physical or social distancing, self-isolation, quarantine and even lockdown of entire territories and communities) are unsustainable, many countries have decided to ease and relax COVID-19-induced restrictions. Since these public health measures may have been lifted too soon, second waves and relapses are rather likely to occur. In order to avoid strained and overwhelmed healthcare facilities as in the early phase of the pandemic, since COVID-19, influenza and other respiratory infections, share unspecific symptoms (including fever, cough, sore throat, fatigue, body aches and headache), immunizing against influenza will be fundamental.

In conclusion, community pharmacists are acquiring more professional standing, and decision- and policy-makers should recognize their increased scope of practice and acknowledge their expanded roles, responsibilities, and duties by means of appropriate juridical provisions.

## References

[1] Michaud C.M. (2009). Global Burden of Infectious Diseases. Encycl. Microbiol..

[2] Jones K.E., Patel N.G., Levy M.A., Storeygard A., Balk D., Gittleman J.L., Daszak P. (2008). Global trends in emerging infectious diseases. Nature.

[3] Greenwood B. (2014). The contribution of vaccination to global health: Past, present and future. Philos. Trans. R. Soc. Lond. B Biol. Sci..

[4] Cockburn T.A. (1961). Eradication of Infectious Diseases. Science.

[5] Plotkin S.L., Plotkin S.A., Plotkin S.A., Orenstein W.A. (2004). A Short History of Vaccination.

[6] Eggertson L. (2010). Lancet retracts 12-year-old article linking autism to MMR vaccines. CMAJ.

[7] Rosselli R., Martini M., Bragazzi N.L. (2016). The old and the new: Vaccine hesitancy in the era of the Web 2.0. Challenges and opportunities. J. Prev. Med. Hyg..

[8] Signorelli C., Odone A., Ricciardi W., Lorenzin B. (2019). The social responsibility of public health: Italy’s lesson on vaccine hesitancy. Eur. J. Public Health.

[9] Bragazzi N.L., Gianfredi V., Villarini M., Rosselli R., Nasr A., Hussein A., Martini M., Behzadifar M. (2018). Vaccines Meet Big Data: State-of-the-Art and Future Prospects. From the Classical 3Is (“Isolate-Inactivate-Inject”) Vaccinology 1.0 to Vaccinology 3.0, Vaccinomics, and Beyond: A Historical Overview. Front. Public Health.

[10] Rosselli R., Martini M., Bragazzi N.L., Watad A., Fluad Effect Working Group (2017). The Public Health Impact of the So-Called “Fluad Effect” on the 2014/2015 Influenza Vaccination Campaign in Italy: Ethical Implications for Health-Care Workers and Health Communication Practitioners. Adv. Exp. Med. Biol..

[11] Mahroum N., Watad A., Rosselli R., Brigo F., Chiesa V., Siri A., Ben-Ami Shor D., Martini M., Bragazzi N.L., Adawi M. (2018). An infodemiological investigation of the so-called “Fluad effect” during the 2014/2015 influenza vaccination campaign in Italy: Ethical and historical implications. Hum. Vaccines Immunother..

[12] Levi M., Sinisgalli E., Lorini C., Santomauro F., Chellini M., Bonanni P. (2017). The “Fluad Case” in Italy: Could it have been dealt differently?. Hum. Vaccines Immunother..

[13] Toffolutti V., McKee M., Melegaro A., Ricciardi W., Stuckler D. (2019). Austerity, measles and mandatory vaccination: Cross-regional analysis of vaccination in Italy 2000–2014. Eur. J. Public Health.

[14] Kuchar E., Ludwikowska K., Marciniak D., Szenborn L., Nitsch-Osuch A. (2017). Public Perception of the Risks Associated with Infectious Diseases in Poland: Ebola and Influenza and Their Impact on the Attitude to Vaccination. Adv. Exp. Med. Biol..

[15] Dudley M.Z., Privor-Dumm L., Dubé È., MacDonald N.E. (2020). Words matter: Vaccine hesitancy, vaccine demand, vaccine confidence, herd immunity and mandatory vaccination. Vaccine.

[16] Signorelli C. (2019). Forty years (1978–2018) of vaccination policies in Italy. Acta Bio-Med. Atenei Parm..

[17] Kata A. (2010). A postmodern Pandora’s box: Anti-vaccination misinformation on the Internet. Vaccine.

[18] Crenna S., Osculati A., Visonà S.D. (2018). Vaccination policy in Italy: An update. J. Public Health Res..

[19] Bragazzi N.L. (2019). Pharmacists as Immunizers: The Role of Pharmacies in Promoting Immunization Campaigns and Counteracting Vaccine Hesitancy. Pharmacy.

[20] Ecarnot F., Crepaldi G., Juvin P., Grabenstein J., Del Giudice G., Tan L., O’Dwyer S., Esposito S., Bosch X., Gavazzi G. (2019). Pharmacy-based interventions to increase vaccine uptake: Report of a multidisciplinary stakeholders meeting. BMC Public Health.

[21] Grabenstein J.D., Guess H.A., Hartzema A.G., Koch G.G., Konrad T.R. (2001). Effect of vaccination by community pharmacists among adult prescription recipients. Med. Care.

[22] Stamboulian D., Soler L., Copertari P., Cordero A.P., Vázquez H., Valanzasca P., Monsanto H., Grabenstein J.D., Johnson K.D. (2018). Policies and practices on pharmacy-delivered vaccination: A survey study conducted in six Latin American countries. Rev. OFIL ILAPHAR.

[23] Sauvageau C., Dubé E., Bradet R., Mondor M., Lavoie F., Moisan J. (2013). Immunization services offered in Québec (Canada) pharmacies. Hum. Vaccines Immunother..

[24] Astier A. (2017). Flu vaccination by community pharmacists. Ann. Pharm. Fr..

[25] Poudel A., Lau E.T.L., Deldot M., Campbell C., Waite N., Nissen L.M. (2019). Review. Pharmacist role in vaccination: Evidence and challenges. Vaccine.

[26] Bartsch S.M., Taitel M.S., DePasse J.V., Cox S.N., Smith-Ray R.L., Wedlock P., Singh T.G., Carr S., Siegmund S.S., Lee B.Y. (2018). Epidemiologic and economic impact of pharmacies as vaccination locations during an influenza epidemic. Vaccine.

[27] Petrelli F., Tiffi F., Scuri S., Nguyen C.T.T., Grappasonni I. (2019). The pharmacist’s role in health information, vaccination and health promotion. Ann. Ig. Med. Preventiva Comunita.

[28] Lankinen K.S., Pastila S., Kilpi T., Nohynek H., Mäkelä P.H., Olin P. (2004). Vaccinovigilance in Europe—Need for timeliness, standardization and resources. Bull. World Health Organ..

[29] FEDERFARMA (2020). Vaccinazioni Antinfluenzali, un Successo la Sperimentazione Nella Carnia. Degrassi: “Ruolo Farmacie Particolarmente Importante”. https://www.Federfarma.it/Edicola/Filodiretto/VediNotizia.aspx?id=20879.

[30] Scarpitta F., Restivo V., Bono C.M., Sannasardo C.E., Vella C., Ventura G., Bono S., Palmeri S., Caracci F., Casuccio A. (2019). The role of the Community Pharmacist in promoting vaccinations among general population according to the National Vaccination Plan 2017–2019: Results from a survey in Sicily, Italy. Ann. Ig..

[31] Della Polla G., Napolitano F., Pelullo C.P., De Simone C., Lambiase C., Angelillo I.F. (2020). Investigating knowledge, attitudes, and practices regarding vaccinations of community pharmacists in Italy. Hum. Vaccines Immunother..

[32] Della Polla G., Pelullo C.P., Napolitano F., Lambiase C., De Simone C., Angelillo I.F. (2020). Knowledge, Attitudes, and Practices towards Infectious Diseases Related to Travel of Community Pharmacists in Italy. Int. J. Environ. Res. Public Health.

[33] Ciliberti R., Gorini I., Gazzaniga V., De Stefano F., Gulino M. (2018). The Italian law on informed consent and advance directives: New rules of conduct for the autonomy of doctors and patients in end-of-life care. J. Crit. Care.

[34] Ciliberti R., Gulino M., Gorini I. (2018). New Italian law about end of life: Self-determination and shared care pathway. Recenti Progressi Medicina.

[35] Pelullo C.P., Di Giuseppe G. (2018). Vaccinations among Italian adolescents: Knowledge, attitude and behavior. Hum. Vaccines Immunother..

[36] Abraham O., Brothers A., Alexander D.S., Carpenter D.M. (2017). Pediatric medication use experiences and patient counseling in community pharmacies: Perspectives of children and parents. J. Am. Pharm. Assoc..

[37] Esposito S., Principi N., Cornaglia G. (2014). Barriers to the vaccination of children and adolescents and possible solutions. Clin. Microbiol. Infect..

[38] Wong C.A., Taylor J.A., Wright J.A., Opel D.J., Katzenellenbogen R.A. (2013). Missed opportunities for adolescent vaccination, 2006–2011. J. Adolesc. Health.

[39] Glatman-Freedman A., Nichols K. (2012). The effect of social determinants on immunization programs. Hum. Vaccines Immunother..

[40] FEDERFARMA (2020). Collaborazione Farmacie con Medici Famiglia e Pediatri Per l’aumento Delle Coperture Vaccinali. https://www.federfarma.it/Edicola/Comunicati-stampa/28-05-2020-17-29-48.aspx.

[41] Bragazzi N.L., Mansour M., Bonsignore A., Ciliberti R. (2020). The Role of Hospital and Community Pharmacists in the Management of COVID-19: Towards an Expanded Definition of the Roles, Responsibilities, and Duties of the Pharmacist. Pharmacy.

